# Significance and amplification methods of the purine salvage pathway in human brain cells

**DOI:** 10.1016/j.jbc.2024.107524

**Published:** 2024-07-02

**Authors:** Mai Sekine, Megumi Fujiwara, Ken Okamoto, Kimiyoshi Ichida, Koji Nagata, Russ Hille, Takeshi Nishino

**Affiliations:** 1 Department of Applied Biological Chemistry, Graduate School of Agricultural and Life Science, The University of Tokyo, Bunkyo, Tokyo, Japan; 2 Department of Pathophysiology, Tokyo University of Pharmacy and Life Sciences, Hachioji, Tokyo, Japan; 3 Department of Laboratory of Morphological Analysis, Nippon Medical School, Bunkyo, Tokyo, Japan; 4 Department of Biochemistry, University of California, Riverside, California, USA; 5 Professor Emeritus, Nippon Medical School, Bunkyo, Tokyo, Japan; 6 University of Tokyo Health Sciences, Tama, Tokyo, Japan

**Keywords:** brain, purine metabolism, salvage, HPRT, XOR, pentose phosphate pathway

## Abstract

Previous studies suggest that uric acid or reactive oxygen species, products of xanthine oxidoreductase (XOR), may associate with neurodegenerative diseases. However, neither relationship has ever been firmly established. Here, we analyzed human brain samples, obtained under protocols approved by research ethics committees, and found no expression of XOR and only low levels of uric acid in various regions of the brain. In the absence of XOR, hypoxanthine will be preserved and available for incorporation into the purine salvage pathway. To clarify the importance of salvage in the brain, we tested using human–induced pluripotent stem cell-derived neuronal cells. Stable isotope analyses showed that the purine salvage pathway was more effective for ATP synthesis than purine *de novo* synthesis. Blood uric acid levels were related to the intracellular adenylate pool (ATP + ADP + AMP), and reduced levels of this pool result in lower uric acid levels. XOR inhibitors are related to extracellular hypoxanthine levels available for uptake into the purine salvage pathway by inhibiting the oxidation of hypoxanthine to xanthine and uric acid in various organs where XOR is present and can prevent further decreases in the intracellular adenylate pool under stress. Furthermore, adding precursors of the pentose phosphate pathway enhanced hypoxanthine uptake, indicating that purine salvage is activated by phosphoribosyl pyrophosphate replenishment. These findings resolve previous contradictions regarding XOR products and provide new insights into clinical studies. It is suggested that therapeutic strategies maximizing maintenance of intracellular adenylate levels may effectively treat pathological conditions associated with ischemia and energy depletion.

Uric acid, the end product of purine catabolism in humans, is generated through a two-step oxidation reaction from hypoxanthine to xanthine and xanthine to uric acid catalyzed by xanthine oxidoreductase (XOR) in various organs (Fig. 1). Blood uric acid levels are maintained by a balance between its production and excretion, with fluctuations influenced by many factors, including genetic, diet, and lifestyle factors. Hyperuricemia is a risk factor for gout, mainly affecting men in the middle age, a period often characterized by vigorous activity (1); it is also associated with renal impairment and cardiovascular disease. However, epidemiological studies have suggested that high uric acid levels reduce the risk of neurodegenerative diseases and may benefit neuroplasticity and cognitive function (2). The pathogenesis of neurodegenerative diseases often involves oxidative stress, and the strong antioxidant effect of uric acid is supported as a mechanism underlying the reduced risk of these diseases (3, 4). Many epidemiological studies have investigated the association between blood uric acid levels and neurological diseases, such as Parkinson’s disease (5, 6, 7), Alzheimer’s disease (8), and amyotrophic lateral sclerosis (9). The association between low uric acid levels and neurodegenerative diseases may be due to the reduced antioxidant capacity of low uric acid levels (10). At present, however, data on the relationship between uric acid levels in human brain tissue and neurological diseases are insufficient. For example, xanthinuria, an XOR deficiency (11, 12), is typically asymptomatic despite extremely low serum uric acid levels (<1 mg/dl), and the mechanism of neuroprotection by uric acid remains unclear.

**Figure 1:**
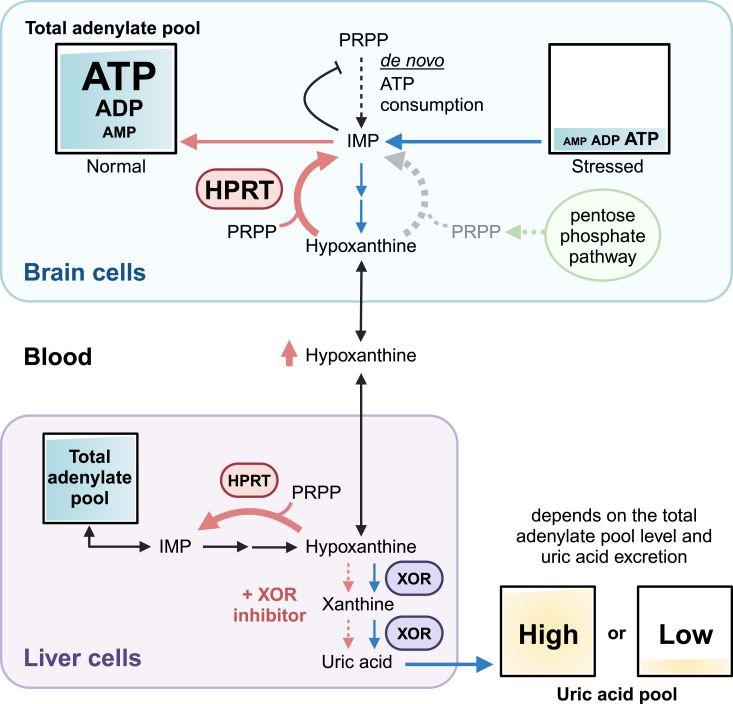
**The bifurcation of hypoxanthine between oxidation by XOR and conversion to IMP by HPRT**. The brain has no XOR and high expression of HPRT, and the increased hypoxanthine is salvaged as a result of ATP consumption. Excess purines are transferred into the blood as purine nucleosides and purine bases, which are metabolized to uric acid in XOR-expressing tissues, mainly the liver. The amount of hypoxanthine that can be stored in the cell is limited; XOR inhibitors increase extracellular hypoxanthine. Hypoxanthine is taken up by purine-deficient cells. PRPP is also a substrate of purine *de novo* pathway, but preferentially enters the salvage pathway when available purine bases are present. The resulting IMP and AMP feedback inhibit the initial step of the *de novo* pathway. PRPP is present in low levels and requires supply by the pentose phosphate pathway to sustain salvage. Total adenylate pool is rapidly reduced by excessive stress and low levels are maintained in persistent stress. These conditions cause variations in the uric acid pool. Created with BioRender.com. HPRT, hypoxanthine phosphoribosyltransferase; XOR, xanthine oxidoreductase.

The xanthine oxidase (XO) form of XOR is thought to be a major source of reactive oxygen species (ROS), and its accumulation has been implicated in pathological mechanisms, such as ischemia-reperfusion injury (13). Therefore, XOR inhibitors, such as allopurinol and febuxostat, have been proposed to exert tissue protective effects by removing ROS (14), but many studies were not consistent. However, a recent study showed that xanthine dehydrogenase (XDH) to XO conversion occurs only in limited cell types expressing both XOR and lactoperoxidase (15), and is likely due to artifacts during isolation and sample preparation in most cases. The characteristic of the inhibitor and the lifetime of the enzyme may also affect the results, as shown with allopurinol (16). Allopurinol is a suicide substrate and its metabolite, oxypurinol, has a weaker inhibitory effect when dissociated from the XOR. This means that multiple doses are required to maintain inhibition, but single doses have also been used in recent years, and it is questionable whether the study design was appropriate. Although the underlying mechanisms need to be investigated, the expression of XOR in the brain is still unclear (17, 18, 19), and no direct analysis has been conducted to determine whether XOR and its substrates, hypoxanthine and xanthine, as well as its product, uric acid, are present in human brain neurons. In contrast, hypoxanthine administration ameliorated brain damage in rabbits (20). The most likely mechanism for XOR inhibitors is not ROS inhibition but energy metabolic effects *via* the purine salvage pathway, mainly through the action of hypoxanthine phosphoribosyltransferase (HPRT) (21, 22, 23, 24). HPRT directly converts hypoxanthine and guanine to IMP and GMP and is expressed in high levels in the brain (25). Hypoxanthine is involved in the purine salvage pathway. Additionally, uric acid precursors, such as inosine and adenine, have been shown to maintain ATP levels, as previously reported in erythrocytes (26). Neural activity requires large amounts of ATP as an energy source. Studies on the ubiquitin-proteasome system using rabbit reticulocytes have shown that a decrease in ATP content reduces the efficiency of overall proteolysis in cells undergoing differentiation into erythrocytes (27). The accumulation of misfolded proteins leads to the formation of aggregates (28). Thus, maintaining ATP levels may conceivably lead to neuroprotection. However, the results obtained from erythrocyte studies cannot be extrapolated to the neural tissue. Mature mammalian erythrocytes lack intracellular organelles and are unable to synthesize new protein, and energy is supplied *via* anaerobic glycolysis. In addition, erythrocytes do not have *de novo* biosynthetic pathways for purine (29), purine pools being maintained solely by the salvage pathway. The brain is similar to erythrocytes in high HPRT expression; however, considerable evidence suggests the presence of the *de novo* pathway (30, 31). Numerous reports based on animal experiments and gene expression information are available; however, these have not been fully verified because of species differences in purine metabolism (32). Therefore, we conducted our experiments using samples of human origin.

The present study finds that uric acid levels are low in human brain tissue, and XOR is not expressed to any significant degree. The results question the long-held view that uric acid is a biologically essential antioxidant. Therefore, a detailed analysis of purine metabolism has been performed using human brain tissue and human induced pluripotent stem (iPS)-derived neurons, focusing on the extent to which HPRT is energetically important for brain neurons. Furthermore, the study investigated the role of XOR inhibitors relevant to the increase in extracellular hypoxanthine levels and explored potential effective additives for maintaining maximal neuronal energetics.

## Results

### Human HPRT expression and activity is high

We examined HPRT activity and expression in erythrocytes (mouse, rat, rabbit, and human) and rabbit reticulocytes. As shown in Fig. S1, the amino acid sequence homology was very high among the species. However, HPRT activity in mice and rats was tens of times lower than that in humans, whereas rabbits were as highly active as humans (Table S1). Furthermore, protein expression levels were correlated with activity. Thus, the expression of purine-metabolizing enzymes differed significantly among animal species. Therefore, experiments with samples of human origin were desirable.

### Uric acid levels are extremely low in human brain tissue; there is no expression of XOR

**Figure 2:**
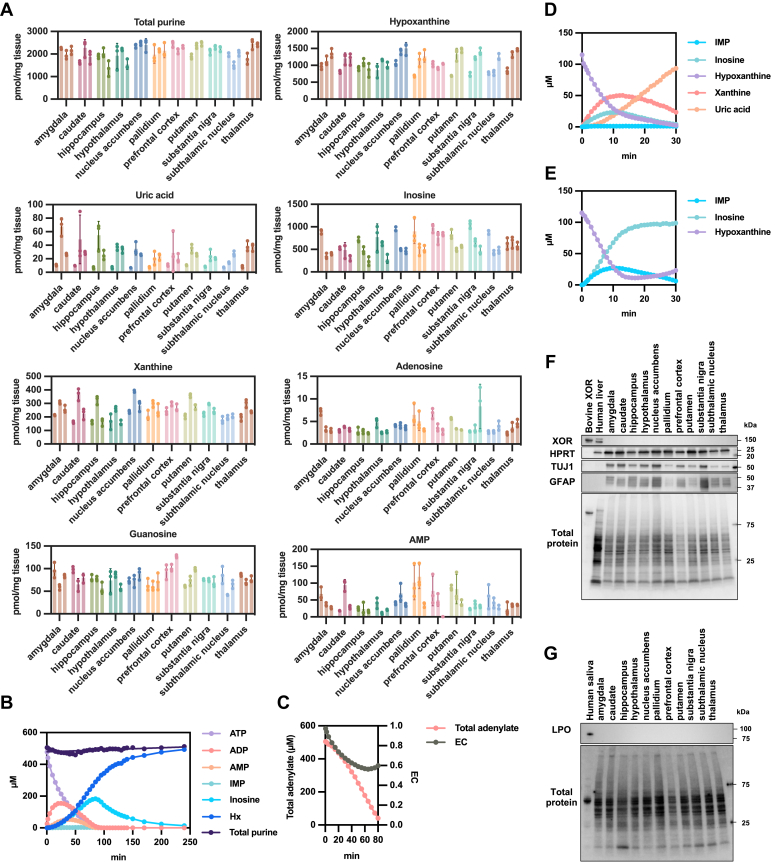
**Purine metabolites in human brain tissue**. *A*, the concentrations of purine metabolites extracted from various regions. Total purine = uric acid + xanthine + hypoxanthine + inosine + adenosine + guanosine + AMP. *B* and *C*, time course of ATP breakdown in dialysis tissue lysate. In addition, 50 mM Tris–HCl buffer pH 7.4, 4.2 mg/ml dialysis human brain tissue lysate, 500 μM ATP, 5 mM MgCl_2_. Energy charge (EC) and total adenylate were calculated as follows: EC = (ATP + 0.5 × ADP)/(ATP + ADP + AMP). Total adenylate = ATP + ADP + AMP. *D* and *E*, time course of hypoxanthine degradation and salvage in dialysis tissue lysate. *D*, 50 mM Tris–HCl buffer pH 7.4, 4.3 mg/ml dialysis rat liver lysate, 500 μM PRPP, 100 μM hypoxanthine, 5 mM MgCl_2_. *E*, 50 mM Tris–HCl buffer pH 7.4, 4.2 mg/ml dialysis human brain tissue lysate, 500 μM PRPP, 100 μM hypoxanthine, 5 mM MgCl_2_. *F* and *G*, Western blotting using extracts prepared from human brain tissue. *F*, detection of XOR, HPRT, TUJ1, GFAP, and total protein in the human brain. Bovine XOR purified enzyme was applied at 0.5 μg/well. Human liver and brain lysates were applied at 10 μg/well. Total protein was stained using No-Stain Protein Labeling Reagent (Thermo Fisher Scientific). *G*, Western blotting of LPO was performed on human brain tissue. Human saliva and brain lysate were applied at concentrations of 1.5 μg/well and 10 μg/well, respectively. EC, energy charge; GFAP, glial fibrillary acidic protein; HPRT, hypoxanthine phosphoribosyltransferase; LPO, lactoperoxidase; TUJ1, βIII-tubulin; XOR, xanthine oxidoreductase.

Purine metabolites were determined in each of several regions of the human brain using HPLC (Fig. 2*A*). ATP and ADP were not present at detectable levels in this experiment; however, AMP, adenosine, inosine, guanosine, hypoxanthine, xanthine, and uric acid were detected. The total purine content was 2000 pmol/mg tissue, comparable to the level of ATP in the rat brain (33), suggesting that ATP degradation occurred in the course of sample preparation. Assuming that XOR were expressed in the human brain, tissues with progressive degradation should have accumulated more uric acid as an end product. However, most of the accumulated purines were hypoxanthine and, to a lesser extent, inosine. Xanthine and guanosine, the breakdown products of GMP, were less abundant. Uric acid accounted for only 1% of the total purines. We then dialyzed the human brain tissue lysate to remove small molecules, and then followed the degradation of added ATP. HPLC was used to analyze the degradation products. As shown in Figure 2, *B* and *C*, hypoxanthine accumulated as an end product, and no uric acid was produced, consistent with the absence of XOR activity. The increase in hypoxanthine levels was pronounced after an increase in AMP levels (Fig. 2*B*). This works in the direction of maintaining energy charge (EC), as shown in Figure 2*C*.

Next, we added hypoxanthine and PRPP, substrates of HPRT to the similarly dialyzed human brain and rat liver lysates to examine metabolic reactions. Degradation to xanthine and uric acid was observed in rat liver lysates expressing XOR (Fig. 2*D*); however, IMP levels did not increase. In contrast, xanthine and uric acid were not produced in the human brain (Fig. 2*E*); however, inosine and IMP were produced at significant levels. Inosine could be produced either by the degradation of the generated IMP or directly from hypoxanthine by purine nucleoside phosphorylase (PNP). Nevertheless, the absence of XOR resulted in elevated hypoxanthine levels, facilitating salvage.

The expression of HPRT and XOR in the human brain was examined using Western blot analysis (Fig. 2*F*). The expression of βIII-tubulin (TUJ1) and glial fibrillary acidic protein (GFAP) was used to confirm the identity of brain tissue samples. We observed differences in the expression levels of TUJ1 and GFAP in each region (Fig. S2), which was consistent with a previous report (34). In contrast, HPRT was shown to be abundant (Figs. 2*F* and S2) while XOR was not detected in the human brain. As a control, bands for XOR were successfully identified in purified bovine milk and human liver (Fig. 2*F*). In addition, no expression of lactoperoxidase (LPO) was detected in the brain (Fig. 2*G*), LPO having been shown to be essential for the conversion of XDH to XO (15).

### Increased hypoxanthine decreases PRPP and metabolites of pentose phosphate pathway

**Figure 3:**
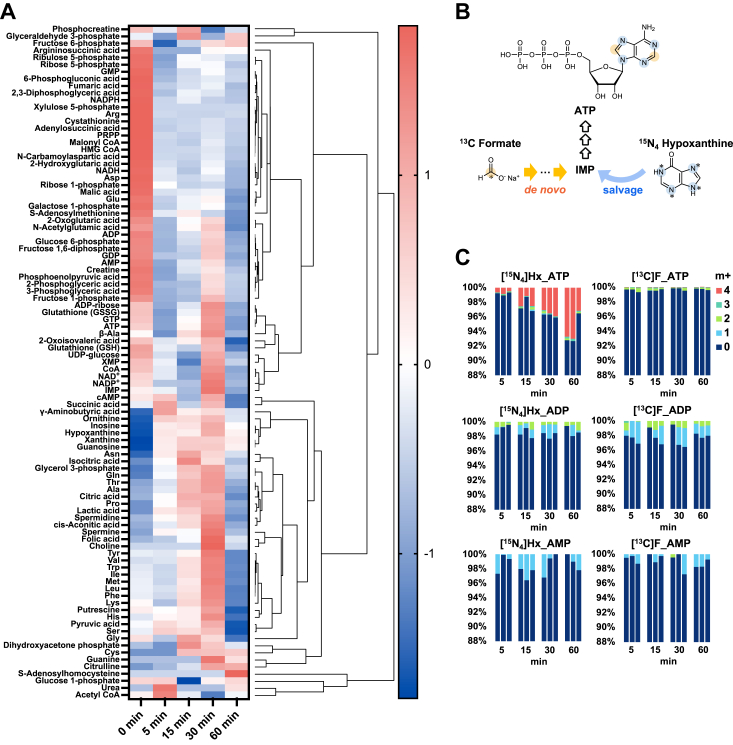
**Purine *de novo* and salvage pathways in neurons derived from iPS cells**. *A*, metabolic change with the addition of hypoxanthine. Subsequently, 100 μM hypoxanthine was added to the culture medium of neurons derived from iPS cells and incubated for 0, 5, 15, 30, and 60 min. Hierarchical clustering was performed using the detected intracellular metabolites and displayed in a HeatMap. *B*, location of incorporation of labeled compounds in ATP. *C*, percentage uptake of the labeled substances for ATP, ADP, and AMP in neurons derived from iPS cells with 50 μM ^15^N4 hypoxanthine or 500 μM ^13^C formate added to the medium. The number indicates the mass number of ^13^C-carbon atoms or ^15^N-nitrogen atoms. iPS, induced pluripotent stem.

Since ATP had been mostly degraded in the human brain tissue samples used above, human iPS neuronal cells were examined to study energy metabolism. Undifferentiated iPS cells were induced to differentiate from neuronal stem cells into neuronal cells, as confirmed by immunocytochemistry and Western blotting (Fig. S3). These iPS neuronal cells were used as brain cell models to examine the metabolic changes caused by the addition of hypoxanthine, using metabolomic analysis (Fig. 3). A heat map also shows that the addition of hypoxanthine affected purine metabolism and other metabolic pathways (Fig. 3*A*). No uric acid was produced, indicating the lack of XOR activity. Intracellular hypoxanthine levels increased over 30 min (Fig. S4) and inosine, xanthine, and guanosine levels also increased. PRPP levels were markedly decreased, consistent with it having served as a substrate for the salvage pathway. Purine nucleotides, such as ATP and GTP, did not increase significantly over time; however, this was likely because the intracellular concentrations were maintained at a steady state. Unfortunately, cyclic AMP could not be elucidated due to technical limitations in this method, yet it may partly contribute to the observed results (35). The metabolites of the pentose phosphate pathway (ribose 1-P, ribulose 5-P, ribose 5-P, 6-phosphogluconate, and xylulose 5-P) were reduced (Fig. S4), again (as with PRPP depletion) consistent with activation of the purine salvage pathway.

### The purine salvage pathway is predominant in iPSC-derived neurons, with hypoxanthine incorporated into ATP

Under the same neuronal cell culture conditions, ^15^N_4_ hypoxanthine or ^13^C formate was added and examined for incorporation into the adenylate. Figure 3*B* shows the isotope-labeling sites demonstrating that label was incorporated into the purine skeleton. The purine skeleton contained two carbon atoms (C-2 and C-8) derived from formate and four nitrogen atoms derived from amino acids *via* the *de novo* biosynthetic pathway (Fig. 3*B*). Therefore, ^13^C formate incorporation yields m+1- or m+2-labeled purine nucleotides. On the other hand, the salvage of ^15^N_4_ hypoxanthine replaces purine bases; thus, m + 4 purine nucleotides were detected. The labeling rates for ATP, ADP, and AMP are shown in Figure 3*C*. The incorporation of ^13^C formate into ATP, ADP, and AMP was observed at low levels, but could not be confirmed to increase over time; however, the addition of ^15^N_4_ hypoxanthine increased m + 4 ATP over time. The confirmation of uptake as early as 5 min suggests that purine salvage activity was much more predominant than purine *de novo* biosynthesis in iPS neuronal cells. Although ^15^N_4_ hypoxanthine incorporation into ADP and AMP could not be confirmed, this was thought to be due to the difficulty of detection because of their lower concentrations than ATP.

### The adenylate pool decreases with exposure to FCCP

**Figure 4:**
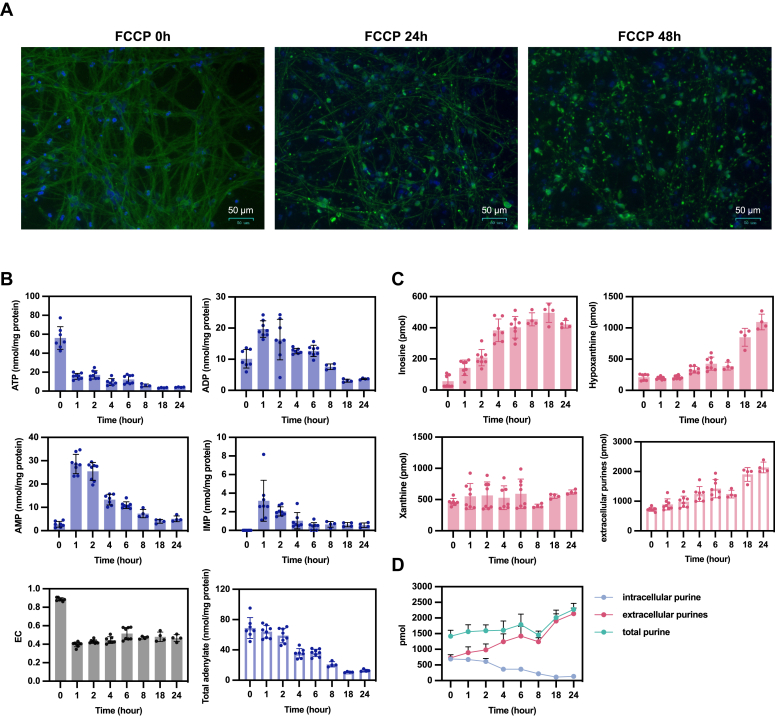
**Neuronal response to uncoupler-induced stress**. *A*, neural differentiation induction from day 14 onward: 10 μg/ml FCCP was added to the culture medium and incubated for 0, 24, and 48 h at 37 °C under 5% CO_2_. After incubation, cells were fixed in 4% paraformaldehyde and stained with TUJ1 and Hoechst 33342. *B* and *C*, intracellular metabolites (*B*) and medium components (*C*) were measured by HPLC over time by adding 10 μg/ml FCCP to the culture medium after neural differentiation induction day 14 and incubating at 37 °C under 5% CO_2_ (n = 4 or more). Total adenylate and EC were calculated. *D*, based on the results of (*B* and *C*), the sum of intracellular purines (ATP + ADP + AMP + IMP) and extracellular purines (inosine + hypoxanthine + xanthine) in the medium is represented graphically as total purine. FCCP, carbonyl cyanide p-trifluoromethoxyphenyl hydrazone; TUJ1, βIII-tubulin.

The above results indicate that hypoxanthine was incorporated into ATP; however, no statistically significant changes were observed at each point from the quantitative results for ATP, ADP, and AMP, and the expected increase in ATP and total adenylate could not be confirmed under normal culture conditions (Fig. S5). We, therefore, added carbonyl cyanide p-trifluoromethoxyphenyl hydrazone (FCCP), a potent mitochondrial uncoupler, to the neuronal culture medium to induce stress. We first examined ATP degradation and its effects during stress. Changes in neuronal morphology and purine metabolite levels induced by FCCP are shown in Figure 4. FCCP induced bead formation on neurites (Fig. 4*A*), consisting of collapsed cytoskeletal and motor proteins resulting from impaired neuronal transport secondary to intracellular energy loss (36). FCCP markedly decreased intracellular ATP levels, whereas ADP, AMP, and IMP levels increased (Fig. 4*B*). The total amount of adenylate derivatives was maintained for up to 2 h but then decreased, while inosine and hypoxanthine in the medium increased (Fig. 4*C*). No significant change in xanthine content in the medium was observed. The sum of the intracellular and medium purines remained constant (Fig. 4*D*). At 24 h, when bead formation was observed, little intracellular total adenylate remained.

**Figure 5:**
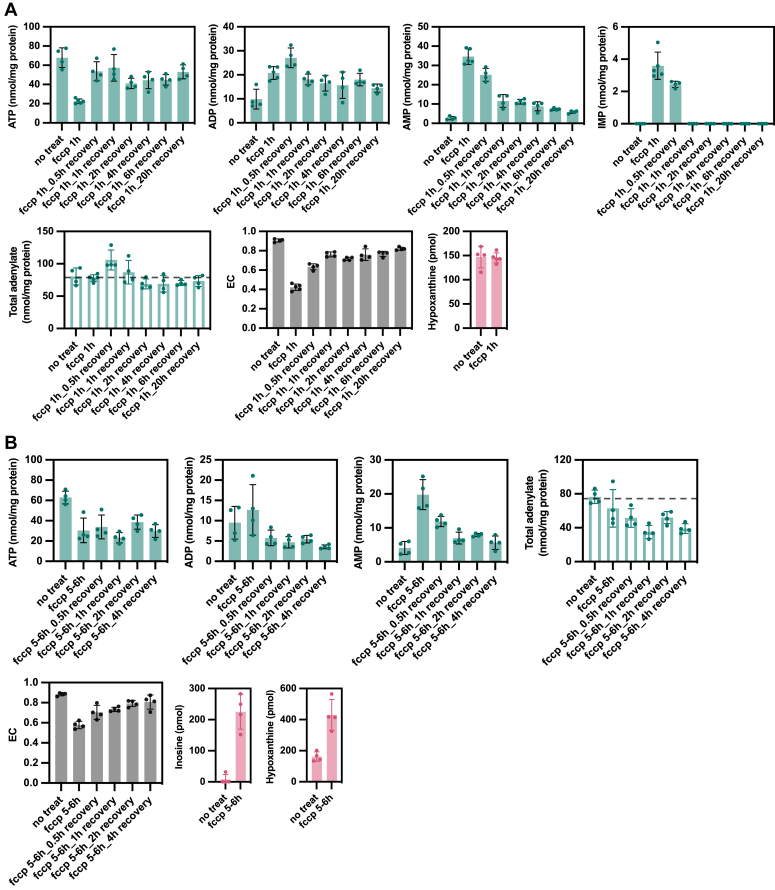
**Changes in purine metabolites during recovery from stress**. *A*, after induction of neuronal differentiation on day 14, 10 μg/ml FCCP was added to the culture medium and incubated under 5% CO_2_ at 37 °C for 1 h. Intracellular metabolites and medium components were measured by HPLC over time after replacement with new culture medium (n = 4 or more). Total adenylate and EC were calculated. *B*, after induction of neuronal differentiation on day 14, 10 μg/ml FCCP was added to the culture medium and incubated for 5 to 6 h at 37 °C under 5% CO_2_. Intracellular metabolites and medium components were measured by HPLC over time after replacement with new culture medium (n = 4 or more). Total adenylate and EC were calculated. FCCP, carbonyl cyanide p-trifluoromethoxyphenyl hydrazone.

After exposure to FCCP, the cells were tested for recovery by replacing the medium with fresh medium (Fig. 5). We also examined whether the recovery of adenylate levels differed depending on the duration of exposure to FCCP. When cells were exposed to FCCP for 1 h (Fig. 5*A*), ATP and EC levels were significantly reduced; however, total intracellular adenylate and hypoxanthine levels in the medium remained unchanged, and only the distribution of adenylate changed. Small amounts of intracellular IMP were also detected. One hour after exchange into FCCP-free medium, the adenylate and EC recovered to almost normal levels. In contrast, 5 to 6 h of FCCP exposure increased the inosine and hypoxanthine levels in the medium (Fig. 5*B*). At 4 h after exchange into FCCP-free medium, EC had mostly recovered, but ATP and total adenylate levels did not return to normal. This indicated a reduction in the purine pool, as hypoxanthine or inosine, which accumulated in the medium due to progressive intracellular purine degradation and was removed upon medium exchange.

### XOR inhibitors afford neuroprotection by preventing further the adenylate pool loss under stress

**Figure 6:**
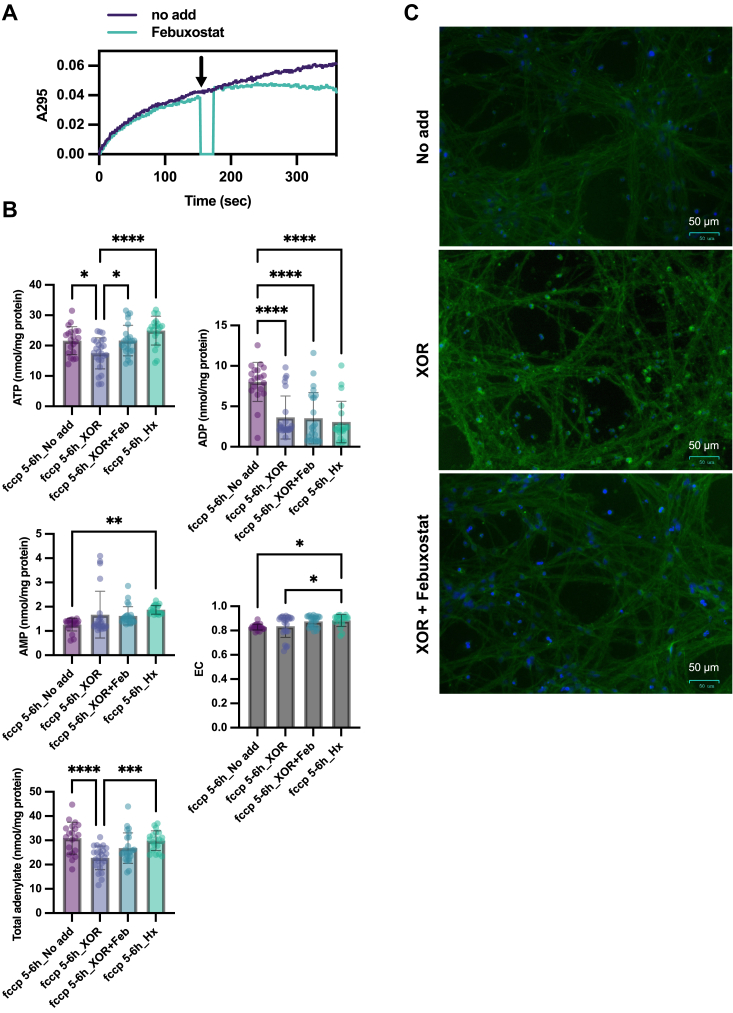
**Effects of XOR during recovery from stress**. *A*, uric acid production was followed at 295 nm by adding 150 μM xanthine and 0.2 μM XOR to nerve culture medium. Uric acid production was inhibited by the addition of 2 μM febuxostat at the *arrows*. *B*, for the induction of neuronal differentiation, 10 μg/ml FCCP was added to the culture medium from day 14 onward and incubated at 37 °C under 5% CO_2_ for 5 to 6 h. After 18 h of incubation with new culture medium, intracellular adenylate was measured by HPLC, and total adenylate and EC were calculated. Medium and PBS (No add, n = 20), 0.2 μM XOR added (XOR, n = 24), 0.2 μM XOR and 2 μM febuxostat (XOR + Feb, n = 24), 20 μM hypoxanthine (Hx, n = 20). One-way ANOVA was used to compare the mean of each column with the mean of every other column. ∗*p* < 0.05, ∗∗*p* < 0.01, ∗∗∗*p* < 0.001, ∗∗∗∗*p* < 0.0001. *C*, cells were fixed in 4% paraformaldehyde and stained with TUJ1 and Hoechst 33342 after incubation under the conditions highlighted in (*B*). FCCP, carbonyl cyanide p-trifluoromethoxyphenyl hydrazone; XOR, xanthine oxidoreductase; TUJ1, βIII-tubulin.

XOR was not expressed in neuronal cells derived from iPS cells. Therefore, we decided to add XOR to the recovery medium to test the neuroprotective effects of the XOR inhibitors. Unlike normal XOR reaction solution condition (*e.g.* Tris buffer and pyrophosphate buffer), addition of XOR to the culture medium resulted in slight inhibition of uric acid production between 0 and 100 s, producing uric acid at 8.3 nmol/nmol/min (Fig. 6*A*). The added XOR was sufficient to convert all hypoxanthine and xanthine in the medium to uric acid during an 18-h incubation. Additionally, sufficient febuxostat was added to inhibit uric acid production (Fig. 6*A*). After 5 to 6 h of FCCP exposure, followed by 18 h of incubation in XOR-containing recovery medium, the total adenylate levels were further reduced (Fig. 6*B*), whereas with febuxostat, total adenylate levels were maintained at the same level as in the medium alone. Under these conditions, the neurons treated with XOR exhibited pronounced bead morphology (Fig. 6*C*), while those that additionally had febuxostat exhibited markedly suppressed bead formation. This clearly indicated a neuroprotective effect of febuxostat by maintaining intracellular adenylate pool. Febuxostat prevented further the adenylate pool loss due to the action of XOR, but did not increase ATP levels to normal levels. Therefore, only hypoxanthine was added to the medium, which was expected to increase total adenylate *via* salvage, but there was no significant difference in ATP and total adenylate compared to the medium alone (Fig. 6*B*).

### ATP is enhanced by pentose supplementation

**Figure 7:**
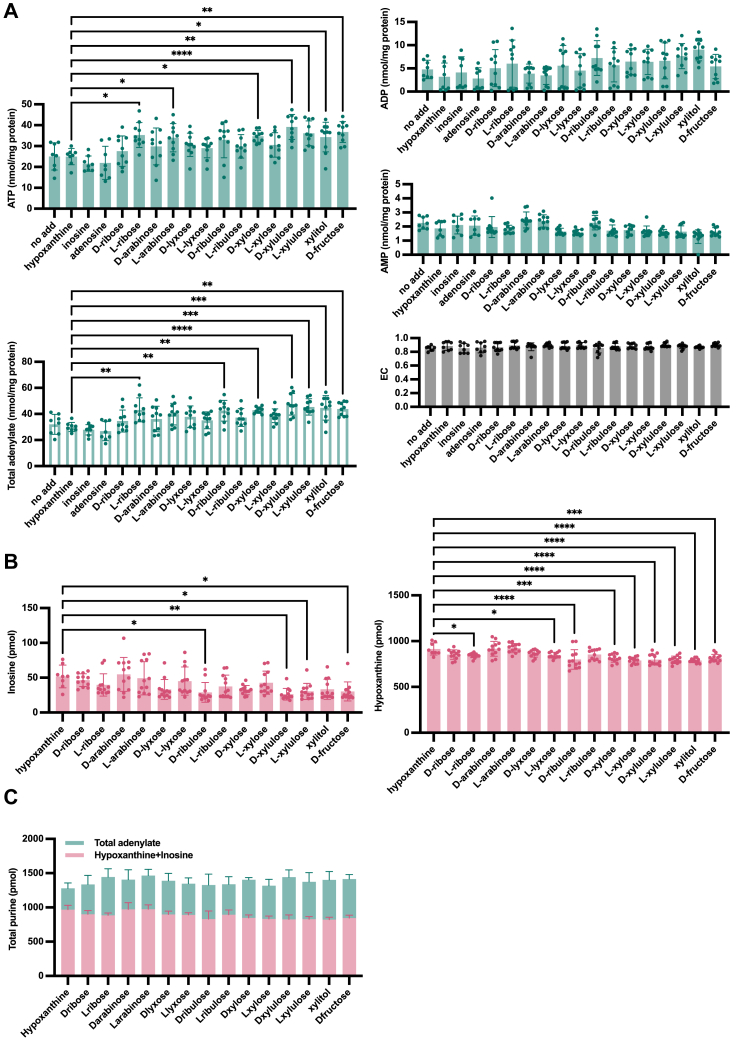
**Effects of pentoses addition on recovery from stress**. *A*, induction of neuronal differentiation from day 14 onward: 10 μg/ml FCCP was added to the culture medium and incubated at 37 °C under 5% CO_2_ for 5 to 6 h. To the new culture medium, 20 μM hypoxanthine and 10 μg/ml (67 μM) various pentoses were added and incubated for 18 h. The intracellular adenylate was measured by HPLC, and total adenylate and EC were calculated (*A*). One-way ANOVA was used to compare the mean of each column with the mean of a hypoxanthine column: no add, inosine, adenosine, and hypoxanthine (n = 8), pentoses, xylitol, and D-fructose (n = 10). ∗*p* < 0.05, ∗∗*p* < 0.01, ∗∗∗*p* < 0.001, ∗∗∗∗*p* < 0.0001. *B*, the same conditions as in (*A*) were used. The medium was collected and measured for hypoxanthine and inosine by HPLC. One-way ANOVA was used to compare the mean of each column with the mean of a hypoxanthine column: hypoxanthine (n = 8), pentoses, xylitol, and D-fructose (n = 12). *C*, sum of results for conditions (*A* and *B*) (pmol). FCCP, carbonyl cyanide p-trifluoromethoxyphenyl hydrazone.

Hypoxanthine was salvaged by HPRT within neurons; however, depletion of PRPP prevented an increase in the adenylate pool above a certain level. Therefore, a precursor thought to be a substrate for the pentose phosphate pathway was added along with hypoxanthine (Fig. 7). Xylitol and fructose, known to increase uric acid levels, were also added with hypoxanthine. Xylitol is taken up by the cell without the assistance of insulin and converted into D-xylulose or L-xylulose. D-xylulose is phosphorylated to D-xylulose 5-phosphate, which enters the pentose phosphate pathway (37). Fructose is phosphorylated by hexokinase and enters the glycolytic pathway as fructose 6-phosphate. It also gives rise to ribose 5-phosphate by reversing the nonoxidative phase of the pentose phosphate pathway using fructose 6-phosphate. Neither XOR nor febuxostat were added to the medium, assuming that the complete inhibition of XOR in the whole metabolic state, and hypoxanthine and pentoses reach neurons through the blood-brain barrier. A pronounced increase in total adenylate content was observed for L-ribose, D-ribulose, D-xylose, D-xylulose, L-xylulose, xylitol, and D-fructose (Fig. 7*A*), which significantly reduced the level of hypoxanthine in the culture medium (Fig. 7*B*). The sum (pmol) of intracellular adenylate and medium purines (hypoxanthine and inosine) was constant (Fig. 7*C*), suggesting uptake of purines from the medium due to the increased salvage activity.

## Discussion

ROS have been implicated in the pathogenesis of neurodegenerative diseases. However, XOR expression in the brain, as well as that of LPO, which is involved in conversion of XDH to the ROS-generating XO form (15), was not detected in Western blots. The contribution of XOR as a source of ROS in the development of neurodegenerative diseases appears unlikely. This is consistent with the fact that XO-locked type mutant (W338A/F339L) knock-in mice showed no differences in neurological development or lifespan, despite overproducing ROS compared to WT mice (38). The ROS-overproducing transgenic mice generate ROS in tissues where XOR is expressed, but not in the brain.

In this study, only small amounts of uric acid were detected in the brain tissue, which in fact may have been derived from blood passing through the cerebral vessels. However, it is not known whether XOR is localized in the microvessels of the human brain. In rats, XOR has been shown to be present in microvessels isolated from the rat brain cortex by homogenization (39), but has been detected in relatively high concentrations in normal rat blood compared to other animal species (19), and there is no reliable evidence. Human erythrocytes do not express XOR, and the small amount of XOR present in the blood is mainly released from the liver and intestine, where it is localized. The possibility that uric acid is transported to the brain tissue *via* the blood-brain barrier and urate transporters remains unclear. However, it is worth noting that uric acid in the brain may be influenced by serum uric acid levels, as evidenced by higher uric acid levels in the cerebrospinal fluid of patients with high serum uric acid levels (40). However, it is unlikely that uric acid in the brain makes an important physiological contribution given the absence of severe neurological symptoms in patients with XOR deficiency (12).

Intracellular purine nucleotide levels are tightly regulated by *de novo*, salvage, and degradation pathways. Excess purines are excreted from the cell as free purine bases and nucleosides, which can pass through the cell membrane. Erythrocytes are involved in the transport of purines between tissues, and purine bases that reach systemic circulation are effectively recycled *via* the salvage pathway. In addition, allosteric feedback inhibition by purine nucleotides of phosphoribosyl pyrophosphate amidotransferase, the rate-limiting enzyme in the purine *de novo* pathway, accounts for the decreased total purine excretion that is observed in patients with XOR deficiency and those taking XOR inhibitors (11, 41). In tissues where XOR is expressed, purine bases are metabolized to uric acid and excreted extracellularly. Overproduction-type hyperuricemia is observed in diseases where the *de novo* pathway is enhanced, such as PRPP synthase superactivity and HPRT deficiency (42, 43).

Under normal conditions, ATP is the predominant intracellular purine. ATP is rapidly and massively consumed due to extreme exercise, alcohol consumption, stress, and pathological conditions, thereby altering the ratios of ATP, ADP, and AMP. Sustained stress promotes purine nucleotide degradation and reduces the intracellular adenylate pool. Thus, early stress alleviation is necessary to maintain adenylate levels, as the individual’s energy needs cannot be met and cell damage may occur if lost purines are not replenished, in extreme cases resulting in cell death. XOR can cause hypoxanthine to be released from the cell as xanthine or uric acid before salvage to IMP occurs, thus reducing the adenylate nucleotide pool (44). The intracellular adenylate nucleotide pool affects blood uric acid levels. Therefore, the relationship between hyperuricemia and neuroprotection based on previous epidemiological studies may reflect the presence of an upstream intracellular adenylate pool, *i.e*., high ATP synthesis capacity or increased ATP degradation. In addition, as the number of viable cells is measured based on the amount of ATP in cultured cells, tissue volume also influences the adenylate nucleotide pool. Serum uric acid levels in men are usually higher than in women, and this difference begins at puberty and is directly correlated with muscle mass (45). Furthermore, uric acid levels have been shown to be causally related to brain volume (46, 47). However, the relationship is not simple and does not imply that high uric acid levels correlated with increased brain volume. Low uric acid levels may represent a reduced adenylate nucleotide pool, which is caused by low nutrition, persistent pathological conditions, such as hypoxia and ischemia, and various age-related functional declines. In such background conditions, there is an imbalance between ATP synthesis and consumption, which may be a factor affecting brain volume. This provides an explanation for many epidemiological results, such as that lower serum uric acid levels are associated with worse motor function in patients with Parkinson’s disease (48) and that a U-shaped relationship has been found between uric acid levels and risk of cardiovascular disease or risk of mortality (49). Therefore, uric acid levels at a single point in time do not accurately represent the state of the body and should be considered carefully.

The principal end product of purine metabolism in human brain tissue was hypoxanthine. Hypoxanthine, increased by energy consumption, is either recycled to adenine nucleotides *via* the salvage pathway or transferred to the bloodstream. During periods of stress, the degradation pathway is active, and salvage is not observed. Since the brain is always metabolically active, conversion to nonreusable uric acid due to the presence of XOR is inefficient. Purine synthesis *via* the *de novo* pathway consumes ATP and may accelerate adenylate degradation under stress conditions. Thus, the absence of XOR in conjunction with high levels of HPRT activity can be considered energy-efficient state, which is of profound significance in the human brain.

Pathological findings similar to Alzheimer’s disease are observed in diseases with an imbalance between *de novo* and salvage pathways. HPRT deficiency, known as Lesch-Nyhan syndrome (LNS), causes hyperuricemia and severe neurological symptoms, including self-injurious behavior. The lack of salvage pathway results in increased metabolism of hypoxanthine to uric acid but also enhanced *de novo* pathway. The pathological anatomy of LNS is marked by brain atrophy due to significant neuronal cell death. In addition, the presence of phosphorylated tau protein has been observed in patients with very mild LNS who have survived to an older age (30 years old) (50). Down’s syndrome patients with trisomy of chromosome 21 have also been shown to be prone to the early onset of Alzheimer’s disease. Three genes (*GARS*, *AIRS*, and *GART*) involved in the *de novo* purine biosynthesis pathway have been mapped to chromosome 21 and excessive purine synthesis gives rise to hyperuricemia, increased plasma adenosine levels, and increased adenosine deaminase activity (51, 52). This imbalance in adenylate levels due to abnormal purine metabolism may prevent the degradation of abnormal proteins *via* the ATP-dependent ubiquitin-proteasome pathway, consequently promoting their accumulation in the cell. At least 300 ATP molecules are hydrolyzed during degradation of one molecule of protein, regardless of its folding status (53). Under these conditions, strategies to enhance ATP may be effective in the treatment of these neurodegenerative conditions, a notion supported by research on conserved blood (26). Previous approaches to increase serum uric acid levels for its antioxidant effects are unlikely to be effective. In fact, the treatment of Parkinson’s disease and multiple sclerosis with inosine to increase uric acid levels has proven unsuccessful (54). On the other hand, the conventional strategy of using antibodies to remove aggregates may reduce ATP consumption and cell damage. Antibody therapy is a treatment approach after aggregates have accumulated, whereas ATP-enhancing therapy is targeted before they accumulate. Therefore, combination therapy is expected to be effective because of the different mechanisms of action.

In the absence of XOR in the brain, the XOR inhibitors must be acting elsewhere outside of the brain to maintain higher levels of extracellular hypoxanthine that can be taken up by the neuronal cells and used in the purine salvage pathway (Fig. 1). Allopurinol, febuxostat, and topiroxostat are currently in clinical use for the treatment of hyperuricemia and gout. Many reports indeed highlight their tissue-protective effects. However, clinical data on the association between XOR inhibitors and neurodegenerative diseases have been inconsistent (55, 56, 57). In our opinion, this is because the optimal dosing regimen and dose-dependency of the inhibitors were not taken into account, and the clinical trials were not properly designed. The present results suggest that XOR inhibitors, which lead to an accumulation of hypoxanthine, are needed for salvage enhancement, but it should be noted that the inhibitory effect on the hypoxanthine to xanthine reaction varies between inhibitors. Allopurinol, which has a purine-like skeleton, is converted to oxypurinol by XOR *in vivo* and is also known to be metabolized by HPRT and PNP. Oxypurinol has also been observed to weakly inhibit the conversion of hypoxanthine to xanthine (16), indicating that this drug may be less effective than febuxostat or topiroxostat (22, 58). Nevertheless, recent studies suggest that increasing the efficacy of allopurinol is possible by using it more frequently and at larger doses (16). The essence of the effect of XOR inhibitors is not only to enhance purine salvage but also to reduce energy costs through the coordination of the *de novo* biosynthetic and salvage pathways. Because the ATP-enhancing effect is systemic, the heart and other organs may also benefit. The results of this study thus suggest that eliminating this ATP-enhancing effect by discontinuing the XOR inhibitors may increase the risk of cardiac arrest as a rebound phenomenon. Allopurinol accumulates as oxypurinol, and the risk incurred may be lower than that of other drugs because the release of XOR inhibition occurs somewhat later.

The substrate concentration primarily regulates the metabolic rate of the purine salvage pathway, and the uptake of purines from the diet or other sources can supply salvage substrates. In addition to inosine and adenine, which are well understood for their effectiveness on red blood cells (26), “umami" taste components, such as IMP and GMP, can also be a source of salvage substrates. The high expression of HPRT in the brain suggests that hypoxanthine is particularly effective (59), indicating that the salvage effect in human may be even more promising in humans than in previous animal models. The blood-brain, blood-CSF, and brain-CSF barriers in HPRT-deficient patients have been reported to be permeable to hypoxanthine and xanthine transport, but not to inosine transport (40). Inosine is also metabolized to hypoxanthine by PNP, and adenosine is expected to have a similar effect, as it is converted to hypoxanthine *via* inosine. However, adenosine may exacerbate symptoms in patients with cardiac disease. Adenine and xanthine are also converted to AMP and XMP but may cause kidney stones, as seen in xanthinuria and adenine phosphoribosyltransferase deficiency.

The results of the metabolomic analysis have suggested that hypoxanthine alone has a limited ATP-enhancing effect due to PRPP depletion. Hypoxanthine release from intracellular IMP has been shown to be strictly dependent on PRPP depletion (44). Several pentoses, xylitol, and D-fructose increase hypoxanthine uptake, with particularly large effects observed for compounds associated with the pentose phosphate pathway, suggesting an increase in PRPP *via* this metabolic route. Previous reports of the effect of fructose, xylitol, and D-xylulose on rat hepatocytes have shown increases in PRPP and ribose 5-phosphate levels (60, 61). The pentose phosphate pathway, a metabolic pathway parallel to glycolysis, converts the starting substrate glucose-6-phosphate to pentose and produces NADPH, which is involved in redox homeostasis (62). Glucose-6-phosphate dehydrogenase, the rate-limiting enzyme in the pentose phosphate pathway, is highly active in the brain and its dysregulation has been implicated in neurodegenerative diseases (63, 64). This suggests that metabolic improvement of the pentose phosphate pathway may be therapeutic.

The present study showed that simultaneous administration of XOR inhibitors, hypoxanthine, and pentose can effectively increase ATP levels to near saturation. This strategy suggests their potential efficacy in treating diseases with pathologies of energy depletion and ischemia, as well as neurodegenerative diseases.

## Experimental procedures

### Human brain tissue

Human brain tissue from three healthy adult male donors (ages 46, 55, and 69 years) was obtained from the Human and Animal Bridging Research Organization in partnership with the National Disease Research Interchange. The study was approved by the ethics committee of Tokyo University of Pharmacy and Life Sciences (approval number: 20–7), and followed the principles outlined in the Declaration of Helsinki. The brain tissue was sliced and divided into 11 regions: amygdala, caudate, hippocampus, hypothalamus, nucleus accumbens, pallidum, prefrontal cortex, putamen, substantia nigra, subthalamic nucleus, and thalamus. Each region was chopped into approximately 50 mg pieces and stored at – 80 °C until use.

### Reticulocytes and erythrocytes

Rabbit reticulocytes were purchased from Promega. Mouse blood samples (three samples) were provided by the Department of Biochemistry and Molecular Biology (Metabolism and Nutrition), Nippon Medical School. Rat blood samples (three samples) were provided by the Laboratory of Veterinary Physiology, University of Tokyo. Rabbit blood samples (three samples) were provided by the Department of Veterinary Pharmacology, Nippon Veterinary and Life Science University. Human blood samples (three samples) were properly collected at the Department of Biochemistry and Molecular Biology (Metabolism and Nutrition), Nippon Medical School, under medical supervision.

### Measurement of HPRT activity in erythrocytes or reticulocyte lysates

Red blood cells were separated by centrifuging the heparin-collected blood, followed by two washes with PBS. Subsequently, they were stored at – 80 °C. The cells were lysed by freezing and rethawing, diluted with twice the volume of distilled water, and after centrifugation the supernatant was collected. Erythrocyte or rabbit reticulocyte lysates were dialyzed overnight at 50 mM Tris–HCl, pH 7.4, 4 °C using a Mini Dialysis Kit with a 1 kDA cutoff (GE HealthCare). Lysates were incubated with 5 mM MgCl_2_, 1 mM PRPP, and 1 mM hypoxanthine and incubated at 25 °C. The reaction solutions collected over time were treated with perchloric acid and neutralized with K_2_CO_3,_ and IMP formation was determined using HPLC.

### Analysis of purine metabolism in human brain tissue

Brain tissue samples were sonicated for 20 to 30 s after addition of 100 μl of 0.2 M perchloric acid per 0.01 g of tissue wet weight and incubated for 30 min on ice and centrifuged (15,000 rpm, 20 min, 4 °C). The supernatant was neutralized with K_2_CO_3_ and centrifuged (15,000 rpm, 5 min, 4 °C). The supernatant was stored at – 80 °C until it was analyzed by HPLC.

Sliced cerebral tissue or rat liver tissue was crushed in a Potter-type homogenizer; after centrifugation at 15,000 rpm, 20 min, 4 °C, the collected supernatant was dialyzed in 50 mM Tris buffer, pH 7.4. Protein quantification was performed, and the lysates were diluted in 50 mM Tris buffer, pH 7.4. Human brain dialysis lysates were incubated with 500 μM ATP and 5 mM MgCl_2_ at 25 °C. The reaction was stopped by adding perchloric acid, and after centrifugation the supernatant was neutralized by adding K_2_CO_3_ and used for HPLC measurements. Human brain and rat liver dialysis lysates were also treated with 100 μM hypoxanthine, 500 μM PRPP, and 5 mM MgCl_2_, and samples processed using the same procedure.

### Purification and activity measurement of XOR

Fresh milk was provided by the Ranch Animal Farm, affiliated with Nippon Veterinary and Life Science University. XOR was purified as previously described (65). XOR activity was measured by following uric acid production at 25 °C under aerobic conditions using a reaction solution consisting of 0.1 M pyrophosphate buffer (pH 8.5), 0.2 mM EDTA, and 150 μM xanthine. The activity was measured at 295 nm using a UV1800 or UV1900i spectrophotometer (Shimadzu).

### Cell culture and neuronal differentiation

Human iPSC line 201B7 (provided by the RIKEN BioResource Center) was cultured on Matrigel-coated culture dishes in Essential 8 Medium at 37 °C in a humified 5% CO_2_ incubator. Differentiation of iPS cells into neural stem cells was induced using PSC Neural Induction Medium (Thermo Fisher Scientific) according to the manufacturer’s protocol. On day 7, neural stem cells (P0) were harvested and expanded in neural expansion medium containing 50% neurobasal medium, 50% advanced Dulbecco's modified Eagle's medium/F12, and neural induction supplement (Thermo Fisher Scientific) on Matrigel.

For neural maturation, neural stem cells were seeded on poly-D-lysine (Thermo Fisher Scientific)-coated culture dishes. After overnight culture, the cells were transferred to a medium consisting of Neurobasal medium, B-27 supplement, CultureOne Supplement, Glutamax, 200 μM ascorbic acid, 10 ng/ml brain-derived neurotrophic factor, 10 ng/ml glial cell line-derived neurotrophic factor, 10 ng/ml NT-3, and penicillin/streptomycin and allowed to differentiate into neuronal cells. Half of the medium was replaced every 3 to 5 days. The expression of pluripotency and neuronal markers was confirmed by Western blotting and immunofluorescence analysis.

### Immunocytochemistry

Adherent cells were rinsed with PBS and fixed with 4% paraformaldehyde for 10 min. They were washed 2 to 3 times with PBS and blocked with blocking buffer (1% bovine serum albumin in PBS with 0.1% Tween 20) for 30 min at room temperature. Primary antibodies were diluted in blocking buffer and incubated with cells overnight at 4 °C. Primary antibodies used were as follows: Oct3/4 (Wako chemicals, 015–27531), Sox2 (Wako chemicals, 012–27541), Nestin (Cell Signaling Technology, #73349), and TUJ1 (GeneTex, GTX130245). Cells were washed three times with PBS and then incubated for 1 h at room temperature with the following secondary antibodies: Goat Anti-Rabbit IgG (H + L), Alexa Fluor 594 (Invitrogen, A11012), and Goat Anti-Rabbit IgG (H + L), Alexa Fluor 488 (Invitrogen, A11008). The cells were washed thrice with PBS and stained with 4′,6-diamidino-2-phenylindole or Hoechst 33342. Cells were imaged using Keyence all-in-one fluorescence microscope (Keyence) or ZOE fluorescent imager (Bio-Rad Laboratories).

### Metabolomics

Using cells cultured 10 days in neuronal differentiation medium, 50 μM hypoxanthine was added to the culture medium and incubated for 0, 5, 15, 30, and 60 min (n = 1 at each time point, five samples total). Metabolites were extracted according to Human Metabolome Technologies (HMT), Inc instructions. Briefly, the culture medium was removed carefully, and 5% (w/w) mannitol in water was added to each cell culture dish (100 mm). The cells were then washed twice to remove any remaining media. The metabolites were extracted using 800 μl of 100% methanol, followed by the addition of 550 μl of internal standard solution provided by HMT. The extract was transferred to filter units provided by HMT and centrifuged at 9100*g*, 4 °C, for 2 to 5 h. The samples were then stored at – 80 °C until they were shipped to HMT for analysis. The filtrate was dried and then resuspended in 50 μl of Milli-Q water for measurement.

Metabolome measurements were performed at HMT, as previously described (66, 67, 68). Cation and anion mode measurements were performed under the conditions outlined in Table S2. For the capillary electrophoresis-time of flight mass spectrometry measurement data, peaks were extracted using the automatic integration software MasterHands, ver. 2.17.1.11 (Keio University) (69). The CE-QqMS data were automatically extracted from the peaks using MassHunter Quantitative Analysis B.06.00 (Agilent Technologies), an automatic integration software. The peak information obtained from each software included the mass-to-charge ratio (*m/z*), peak area value, and migration time (MT). The obtained peak area values were converted to relative area values using the following formula: area of the target peak/[area of the internal standard × sample volume]. The examined peaks were closely matched and aligned between samples based on *m/z* and MT values. The detected peaks were matched, and compounds registered in the HMT Metabolite Library were searched based on *m/z* and MT values. Tolerances for the search were ± 0.5 min for MT and ± 10 ppm for *m/z*. The mass error (ppm) was calculated using the following formula: [measured value–theoretical value]/measured value × 10^6^. The relative area values obtained were converted into absolute quantitation values using standards. Peak area values corrected using internal standards were used for quantitative conversion, and a calibration curve consisting of three points was created for each metabolite to calculate its concentration. Hierarchical clustering analysis was performed using statistical analysis software developed by HMT. Data were preprocessed by standardizing the data on a per peak basis (μ = 0, σ = 1).

### Isotopomer analysis

Neuronal cells were cultured in the neuronal maturation medium for 10 days. Subsequently, either 500 μM ^13^C formate or 50 μM ^15^N_4_ hypoxanthine (Cambridge Isotope Laboratories) was added to the culture medium and incubated for 5, 15, 30, and 60 min (n = 3 at each time point, 24 samples total). Metabolites were extracted according to HMT, Inc instructions. Briefly, the culture medium was removed carefully, and 5% (w/w) mannitol in water was added to each 100 mm cell culture dish. The cells were washed twice to remove media. The metabolites were extracted using 800 μl of 100% methanol, followed by the addition of 550 μl of internal standard solution provided by HMT. The extract was transferred to filter units provided by HMT and centrifuged at 9100*g*, 4 °C, for 2 to 5 h. The samples were then stored at – 80 °C until they were shipped to HMT for analysis. The filtrate was dried and then resuspended in 50 μl of Milli-Q water for measurement.

The cation and anion mode measurements were performed under the conditions outlined in Table S3. Peaks detected by capillary electrophoresis-time of flight mass spectrometry were automatically extracted using the automatic integration software MasterHands, ver. 2.17.1.11 (Keio University), for peaks with a signal-to-noise (S/N) ratio of three or higher. The software provided the mass-to-charge ratio (*m/z*), peak area, and MT. The obtained peak area values were converted to relative area values using the following formula: area of the target peak/[area of the internal standard × sample volume]. Because these data contained adduct ions, such as Na^+^ and K^+^ and fragment ions due to dehydration and loss of ammonia, these molecular weight-related ions were removed. However, because substance-specific adducts and fragments were also present, it was not possible to scrutinize them all. Peaks that were scrutinized were matched and aligned between samples based on *m/z* and MT values. In addition, the target substances and their stable isotopes registered in the HMT Metabolite Library were matched and searched. Tolerances for the search were ± 0.5 min for MT and ± 30 ppm for *m/z*. The mass error (ppm) was calculated using the following formula: [measured value-theoretical value]/measured value × 10^6^. The mass error (ppm) was calculated using the following formula: (measured value-theoretical value)/measured value × 10^6^. Calibration curves were constructed using peak areas corrected for internal standards, and concentrations were calculated as a single point calibration of 100 μM for each substance (200 μM for the internal standard).

### Measurement of purine metabolites

The purine metabolite levels were determined using a Shimadzu HPLC system. The Shimadzu HPLC system consisted of a system controller (CBM-20A), pump (LC-20AD), autosampler (SIL-20AC, set at 15 °C), column oven (CTO-20A, set at 40 °C), and diode array detector (SPD-M20A). The peak analysis software used was LC Solution. A SUPELCOSIL LC-18-T column (25 cm x 4.6 mm i.d., 5 μm particle size) and a SUPELCOSIL LC-18-T Supelguard guard column were used to separate purine metabolites. Samples of 20 to 60 μl were injected. The flow rate was 0.–0.8 ml/min. The mobile phases were as follows: (A) 0.1 M potassium phosphate buffer and 4 mM tetrabutylammonium hydrogen sulfate (pH 5.5) and (B) 70% buffer A and 30% methanol (v/v). Gradient conditions were set as follows: 0 min (0% B), 2 min (0% B), 4 min (30% B), 12 min (60% B), 15 min (100% B), 19 min (100% B), 20 min (0% B), and 30 min (0% B). The detection wavelengths were 254, 268, and 295 nm. Purines were identified based on their retention times and quantified using external standards. The data were analyzed using LabSolutions, ver. 5.97 SP1.

### Western blot analysis

The brain tissue samples were washed twice with PBS to remove blood and homogenized in N-PER buffer (Thermo Fisher Scientific) mixed with a complete EDTA-free protease inhibitor cocktail (Sigma-Aldrich) using a BioMasher II (Nippi). The homogenates were incubated on ice for 10 min, followed by centrifugation (10,000*g*, 10 min, 4 °C) and collection of the supernatant. Samples were stored at – 80 °C and used for Western blotting. The cultured cells were washed twice with PBS and treated with TrypLE Select. The collected cells were washed with PBS and sonicated in PBS containing a protease inhibitor cocktail.

Protein quantification was performed using the Bradford Protein Assay Kit (TaKaRa Bio). Protein extracted samples were diluted in Laemmli sample buffer (Bio-Rad Laboratories) containing 2-mercaptoethanol and denatured by heating at 95 °C for 5 min. The samples were loaded onto 4 to 20% Mini-PROTEAN TGX Gels (Bio-Rad Laboratories). The gels were run on a Mini-PROTEAN Tetra system at 200 V for 30 min with 1 × Tris/glycine/SDS running buffer. The gel was transferred to a 0.2 μm polyvinylidene fluoride membrane using the Trans-Blot Turbo Transfer System (Bio-Rad Laboratories). The transferred membrane was stained with No-stain Protein Labeling Reagent (Thermo Fisher Scientific) according to the manufacturer’s protocol. The membrane was blocked using EveryBlot blocking buffer (Bio-Rad Laboratories). Primary antibodies were diluted in EveryBlot blocking buffer and applied overnight at 4 °C. The secondary antibody was diluted in EveryBlot blocking buffer and incubated for 1 h at room temperature. After incubation with each antibody, the membrane was washed thrice with Tris-buffered saline with Tween 20. Chemiluminescence development was performed using the Clarity Western ECL substrate (Bio-Rad Laboratories). Western blotting images were captured using an iBright FL1500 Imaging System (Thermo Fisher Scientific). The protein expression levels were normalized to the total amount of loaded protein. Full-length blots are presented in the Supplementary Information. The primary antibodies used were as follows: Oct3/4 (Wako Chemicals, 015–27531), Sox2 (Wako Chemicals, 012–27541), HPRT1 (Proteintech, 15059-1-AP), Xanthine oxidase (Santa Cruz Biotechnology, sc-398548), LPO (Invitrogen, PA5-115521), TUJ1 (GeneTex, GTX130245), GFAP (Cell Signaling Technology, #3670), and Nestin (Cell Signaling Technology, #73349). The secondary antibodies included Donkey Anti-Rabbit IgG H&L (Alexa Fluor 647) (Abcam, ab150075), and Goat Anti-Mouse IgG (H + L) HRP Conjugate (Bio-Rad Laboratories, #1706516).

### Statistical analysis

Statistical analyses were performed using GraphPad Prism 10, version 10.2.0 (https://www.graphpad.com). We used one-way ANOVA for comparisons among groups. *p* < 0.05 was adopted to declare statistical significance. All data were presented as mean ± SD. The specific analytic methods used in each experiment are described in the figure legends.

## Data availability

Please contact the authors for data requests.

## Supporting information

This article contains supporting information.

## Conflict of interest

T. N. and K. O. participated as the inventors of two patents (PCT/JP2016/055226 and PCT/JP2017/006007: dementia such as Alzheimer’s disease). The other authors declare that they have no conflicts of interest with the contents of this article.
