# Supplementary material for: Significance and amplification methods of the purine salvage pathway in human brain cells
Source: J Biol Chem. 2024 Jul 2;300(8):107524. doi: 10.1016/j.jbc.2024.107524 (PMC11342100; doi:10.1016/j.jbc.2024.107524)
Supplement: Supplementary data [file mmc1.docx]

**SUPPLEMENTAL INFORMATION**

**Supplementary Table 1.** **HPRT activity and expression.**

|  | Relative HPRT activity | SEM | N |
| --- | --- | --- | --- |
| Rabbit erythrocyte | 2.021 | ±0.112 | 3 |
| Rabbit reticulocyte | 1.944 | ±0.132 | 3 |
| Human erythrocyte | 1.000 | ±0.029 | 3 |
| Mouse erythrocyte | 0.018 (1/56) | ±0.003 | 3 |
| Rat erythrocyte | 0.048 (1/21) | ±0.010 | 3 |

|  | Relative HPRT expression | SEM | N |
| --- | --- | --- | --- |
| Rabbit erythrocyte | 1.210 | ±0.077 | 1 |
| Rabbit reticulocyte | 1.735 | ±0.247 | 1 |
| Human erythrocyte | 1.000 | ±0.085 | 1 |
| Mouse erythrocyte | 0.010 (1/100) | ±0.001 | 1 |
| Rat erythrocyte | 0.051 (1/20) | ±0.004 | 1 |

**Supplementary Table 2. Measurement conditions for the cation and anion modes.**

|  | Agilent CE-TOFMS system (Agilent Technologies Inc.) | CE: Agilent CE system  MS: Agilent 6460 TripleQuad LC/MS Machine No. QqQ1 |
| --- | --- | --- |
| Capillary: | Fused silica capillary i.d. 50 µm×80 cm | |
| Condition: | Cation mode | Anion mode |
| Run buffer: | Cation Buffer Solution  (p/n: H3301-1001) | Anion Buffer Solution  (p/n: H3302-1021) |
| Rinse buffer: | Cation Buffer Solution  (p/n: H3301-1001) | Anion Buffer Solution  (p/n: H3302-1021) |
| Sample injection: | Pressure injection 50 mbar,  5 sec | Pressure injection 50 mbar,  25 sec |
| CE voltage: | Positive, 30 kV | 30 kV |
| MS ionization: | ESI Positive | ESI Positive and negative |
| MS capillary voltage: | 4,000 V | 4,000 V for positive and  3,500 V for negative mode |
| MS scan range: | *m/z* 50 - 1,000 |  |
| Sheath liquid: | HMT Sheath Liquid  (p/n: H3301-1020) | HMT Sheath Liquid  (p/n: I3300-1030) |

**Supplementary Table 3. CE-TOF-MS measurement conditions for isotope analysis.**

|  | Agilent CE-TOFMS system (Agilent Technologies Inc.) | |
| --- | --- | --- |
| Capillary: | Fused silica capillary i.d. 50 µm×80 cm | |
| Condition: | Cation mode | Anion mode |
| Run buffer: | Cation Buffer Solution  (p/n: H3301-1001) | Anion Buffer Solution  (p/n: H3302-1023) |
| Rinse buffer: | Cation Buffer Solution  (p/n: H3301-1001) | Anion Buffer Solution  (p/n: H3302-1023) |
| Sample injection: | Pressure injection 50 mbar,  10 sec | Pressure injection 50 mbar,  22 sec |
| CE voltage: | Positive, 30 kV | Positive, 30 kV |
| MS ionization: | ESI Positive | ESI Negative |
| MS capillary voltage: | 4,000 V | 3,500 V |
| MS scan range: | *m/z* 50 - 1,000 | *m/z* 50 - 1,000 |
| Sheath liquid: | HMT Sheath Liquid  (p/n: H3301-1020) | HMT Sheath Liquid  (p/n: H3301-1020) |

**Supplementary Fig. 1.** **HPRT amino acid sequences.**

Multiple sequence alignment of human, rabbit, mouse, and rat HPRT amino acid sequences. The same sequences (blue background), different sequences (white background), and basic amino acids (red text) are shown.

**Supplementary Fig. 2.** **Western blotting using extracts prepared from human brain tissue.**

Three samples were taken from each region (11 regions: amygdala, caudate, hippocampus, hypothalamus, nucleus accumbens, pallidum, prefrontal cortex, putamen, substantia nigra, subthalamic nucleus, and thalamus) in healthy donors (n = 3). **A-E** Detection of XOR (A), LPO (B), HPRT (C), GFAP (D), and TUJ1 (E). LPO detection of donor 1 was performed at n = 2 due to lack of subthalamic nucleus sample; E is an empty lane. Bovine XOR purified enzyme and human saliva was applied at 0.5 μg/well and 1.5 µg/well, respectively. Human liver and brain lysates were appliqued at 10 µg/well. The XOR, HPRT, TUJ1, and GFAP image panels in Fig. 2F are derived from the same source image as the “1–2” image placed in the (A), (C), (E), and (D), respectively. The LPO image panel in the Fig. 2G is derived from the same source image as the “3–2” image placed in the (B). **F-H** Signal intensity of HPRT (F), GFAP (G), and TUJ1 (H) bands normalized by total protein.

**Supplementary Fig. 3. Neural differentiation and characterization of iPS cells.**

**A** Expression of Oct4, SOX2, Nestin, and TUJ1 at the stage of differentiation to neurons by western blotting. **B** Immunocytostaining for Oct4, SOX2, Nestin, and TUJ1 at the stage of differentiation to neurons.

**Supplementary Fig. 4. Purine *de novo* and salvage pathways in neurons derived from iPS cells.**

Metabolic changes with addition of hypoxanthine. 100 µM hypoxanthine was added to the culture medium of neurons derived from iPS cells and incubated for 0, 5, 15, 30, and 60 min.

**Supplementary Fig. 5. Quantification results of ATP, ADP, and AMP.**

**A-B** 50 µM ^15^N4 hypoxanthine (A) or 500 µM ^13^C formate (B) added to the medium of iPS cell-derived neuronal cell and incubated for 5, 15, 30, 60 min (n = 3 at each point). Energy charge (EC) and total adenylate were calculated as follows: EC = (ATP + 0.5 × ADP)/ (ATP + ADP + AMP). Total adenylate = ATP + ADP + AMP. One-way ANOVA was used to compare the mean of each column with the mean of every other column. There were no statistically significant differences.
